# Paediatric palliative screening scale as a useful tool for clinicians’ assessment of palliative care needs of pediatric patients: a retrospective cohort study

**DOI:** 10.1186/s12904-021-00765-8

**Published:** 2021-05-24

**Authors:** In Gyu Song, Seung Yeon Kwon, Yoon Jung Chang, Min Sun Kim, Sung Hoon Jeong, Seung Min Hahn, Kyu Tae Han, So-Jung Park, Jin Young Choi

**Affiliations:** 1 Department of Pediatrics, Korea University Guro Hospital, Seoul, Republic of Korea; 2 National Hospice Center, National Cancer Center, 323 Ilsan-ro, Ilsandong-gu, Goyang-si, Gyeonggi-do Republic of Korea; 3 Palliative Care Center, Yonsei Cancer Center, Yonsei University Health System, Seoul, South Korea; 4 Department of Pediatric Hematology-Oncology, Yonsei Cancer Center, Yonsei University Health System, Seoul, Republic of Korea; 5 Department of Pediatrics, Seoul National University Children’s Hospital, Seoul, Republic of Korea; 6 Department of Public Health, Graduate school, Yonsei University, Seoul, Republic of Korea; 7 Department of Pediatrics, Yonsei University College of Medicine, Yonsei University Health System, Seoul, Republic of Korea

**Keywords:** Palliative care, Pediatrics, Pediatrician, Prognosis

## Abstract

**Background:**

Although the importance of palliative care in pediatric patients has been emphasized, many health care providers have difficulty determining when patients should be referred to the palliative care team. The Paediatric Palliative Screening Scale (PaPaS) was developed as a tool for screening pediatric patients for palliative care needs. The study aimed to evaluate the PaPaS as a reliable tool for primary care clinicians unfamiliar with palliative care.

**Methods:**

This was a retrospective cohort study of patients referred to the pediatric palliative care teams in two tertiary hospitals in the Republic of Korea between July 2018 and October 2019.

**Results:**

The primary clinical and pediatric palliative care teams assessed the PaPaS scores of 109 patients, and both teams reported a good agreement for the sum of the PaPaS score. Furthermore, the PaPaS scores correlated with those obtained using the Lansky performance scale. Although the mean PaPaS score was higher in the pediatric palliative care team, the scores were higher than the cut-off score for referral in both groups.

**Conclusion:**

The PaPaS can be a useful tool for primary care clinicians to assess the palliative care needs of patients and their families.

## Background

The World Health Organization defines palliative care as the prevention and relief of physical, psychological, social, and spiritual suffering of adult and pediatric patients and their families due to life-threatening illnesses [1]. Although children’s suffering was not fully recognized for a long time, and the concept of holistic suffering in pediatric patients appeared later than in adults, there has been remarkable progress in pediatric palliative care (PPC) in the past decades due to the efforts of dedicated professionals [2, 3]. However, some barriers still hinder PPC development in several countries and various medical settings [4–7]. Previous studies have indicated that a significant gap exists between the needs of children and their families and the services they receive; one of the main underlying reasons for this is an insufficient understanding of PPC by physicians [8–11]. Therefore, referrals to palliative care are often made late in the disease trajectory even in countries with comparatively better PPC availability [12, 13].

Bergstraesser et al. proposed that palliative care can have more diverse aims in children unlike in adults. These include reducing burdensome treatments in advanced cancer, enhancing the quality of life through effective symptom control, and easing the emotional burden on parents and family. An instrument must be available that captures children’s palliative care needs at an earlier stage than in adults [13]. Thus, they developed a tool for screening patients called the Paediatric Palliative Screening Scale (PaPaS) which aimed to support health care professionals in accurately identifying children with palliative care needs. Previous validation of the tool showed promising results, and additional studies on the PaPaS have been reported [7, 13–15]. However, there are few studies on the clinical application and usefulness of the PaPaS in various settings.

In South Korea, due to the perception and socio-cultural trend among health care providers and the general public, the focus has been on intensive curative treatment rather than the quality of life of seriously ill children. Moreover, PPC development has been slow over the past few decades and is only available in limited settings [16]. The Ministry of Health and Welfare started a pilot project for consultant-based PPC in September 2018 to establish and develop PPC as a national health care policy for children with life-threatening or life-shortening illnesses in South Korea. This project was executed to promote PPC through financial and policy support to PPC providers and monitor the palliative care service delivery processes and their outcomes to apply them to future nationwide policies. During the pilot project, to identify children in need of palliative care, primary health care providers were instructed to use the PaPaS to evaluate children and their families’ palliative care needs and refer them to the PPC team based on the results.

Therefore, we aimed to investigate the agreement between primary care clinicians of the referral and PPC teams in measuring each palliative care needs domain included in the PaPaS. Ultimately, we wanted to investigate whether the PaPaS can be a useful tool for primary referring clinicians who are not as familiar as the PPC team with the palliative care needs of children and their families.

## Methods

### Setting

The PaPaS was developed to assess the palliative care needs of children and improve the timely identification of children who can benefit from a palliative care approach. It consists of five domains: the trajectory of disease and impact on daily activities; expected outcomes of disease-directed treatment and burden of treatment; symptom burden; preferences of the patient, parents, or health care professionals; and estimated life expectancy. A score ≥ 15 indicates that PPC can be initiated [13, 14].

Before beginning the national pilot program, we developed the Korean version of the PaPaS with permission from the original author. Translation, back-translation, and cognitive interviews with experts in palliative care were performed during the translation process. Since the scale was developed for children between 1 and 18 years old, some domains of the PaPaS were difficult to apply in patients < 1 year old [13]. As there is no validated tool for assessing the palliative care needs of patients < 1 year old, we excluded four domains (1.1, 1.2, 2.2, 3.2) from the scale which were not appropriate for infants and developed a modified version for this age group after discussion with the author of the PaPaS. In the pilot program, the criterion for registration and initiating palliative care was set as an index score of 15 for patients ≥1 year old, as recommended by the original study, and 10 for patients < 1 year old according to the modification. Primary care clinicians and the PPC team assessed the patient separately within a day of the consultation.

### Design

We conducted a retrospective cohort study of patients referred to the PPC team. In Korea, palliative care was a very unfamiliar concept, and only a few doctors were confident with it [17, 18]. Therefore, we assumed that primary care physicians had a lack of knowledge in PPC. However, PPC specialists in those teams were required to complete 76 h of palliative care curriculum before participating in the pilot program. The PaPaS scores of the primary clinical team were compared with those of the PPC team. During the development process of the PaPaS, assessing the performance status of patients was considered an important factor in investigating the prognosis and palliative needs of patients and families [13]. Therefore, the scale was compared with the Lansky performance scale that measured the performance status of patients < 16 years old at the same time as the PaPaS evaluation by the PPC team [19].

### Data collection and analysis

The study population comprised patients referred to the PPC team at Seoul National University Children’s Hospital and Severance Children’s Hospital between July 2018 and October 2019. Among them, patients assessed using the PaPaS by both the primary clinical and PPC teams were included. Data were collected from the patients’ hospital records to analyze the clinical characteristics and PaPaS.

Cohen’s Kappa was used to measure the agreement between two raters, and linear regression was performed to assess the correlation between the PaPaS and the Lansky performance scale. The total scores and score of each domain were compared between two raters using a paired t-test. We performed subgroup analyses according to the primary care physicians’ category: medical specialists or residents. For estimating the sensitivity and specificity of PaPaS assessment by the primary clinical team, we compared it with the PPC team to determine whether the sum of the individual domain scores met the criteria for registration. STATA version 15.1 (StataCorp, College Station, TX, USA) was used for statistical analysis.

## Results

A total of 467 patients were referred to the PPC team at both hospitals between July 2018 and October 2019. Among them, 109 patients (23.3%) had the PaPaS assessed by both the primary clinical and PPC teams. The median age was 8 years, and over 60% were boys. In hospital A, 80.0% of subjects were cancer patients, whereas 72.2% were non-cancer patients in hospital B (*p* < 0.001). The performance scale and PaPaS scores were not statistically different between the two hospitals. The mean PaPaS scores of patients aged < 1 and ≥ 1 year old were 14.2 (±4.0) and 22.0 (±5.3), respectively (Table 1).

**Table 1:** Demographics and PaPaS scales of the patients

	*N* = 109
Age at consultation	
Median age in years (IQR)	8 (12)
Age range in years	0 to 23
Patients ≥1 year old (%)	92 (84.4)
Sex (n, %)	
Female	41 (37.6)
Male	68 (62.4)
Type of diagnosis (n, %)	
Cancer	59 (54.1)
Non-cancer	50 (45.9)
Performance scale (mean, SD)	
Lansky	39.9 (22.6)
PaPaS (mean, SD)	
Patients < 1 year old	14.2 (4.0)
Patients ≥1 year old	22.0 (5.3)

*Abbreviations:* PaPaS Scale, Paediatric Palliative Screening Scale, *IQR* interquartile range, *SD* standard deviation

The mean scores were not different between cancer and non-cancer patients (22.8 vs. 21.5, *p* = 0.271). Most patients (93.6%) were referred from the department of pediatrics. Two pediatricians and two nurses were assessors on the PPC team, and their median duration of experience in pediatrics was 9.5 years. There were 55 patients (50.5%) who were assessed the PaPaS by pediatric specialists on the primary clinical teams, and the rest were assessed by pediatric residents (Table 2).

**Table 2:** Demographics of pediatric palliative care and primary clinical teams

Pediatric palliative care team	
Pediatric Palliative Specialist (n)	2
Nurse (n)	2
Career in pediatrics (median)	9.5 (range, 8–12) years
Primary clinical team (n, %)	
Department of Pediatrics	102 (93.6)
Medical specialists	55 (50.5)

Good agreement (kappa = 0.546) was observed for the total PaPaS scores of patients aged ≥1 year between the primary clinical and PPC teams. Medical specialists showed better agreement compared to residents (kappa = 0.673 vs. 0.442). Additionally, the PaPaS scores correlated with those obtained using the Lansky performance scale. The total PaPaS scores and Q1.1 scores were used to assess patients’ performance, correlated with the score calculated by both teams (Table 3).

**Table 3:** Correlation between PaPaS and the Lansky performance scale

PaPaS	Palliative care team	Primary clinical team
Sum		
R-squared	0.402	0.421
ß	−2.676	−2.214
Q1.1 (about performance)		
R-squared	0.326	0.289
ß	−10.503	−8.915

*Abbreviation: PaPaS Scale* Paediatric Palliative Screening Scale

Although the mean PaPaS score was higher in the PPC team, the scores were > 15 in both groups for patients aged ≥1 year. In subgroup analysis, the mean score of medical specialists was comparable with the PPC team (22.3 ± 5.1 vs. 22.3 ± 6.8, *p*-value = 1.0). However, pediatric residents showed a lower mean score than the PPC team (18.8 ± 6.3 vs. 21.7 ± 5.5, p-value < 0.001). Five out of nine sub-scales were statistically different between the two groups, and the scores were mostly higher in the PPC team. For patients aged < 1 year, the total scores (mean) were not significantly different between the two groups and two out of seven sub-scales were different (Table 4). Comparing the total PaPaS scores between the primary clinical and PPC teams, the PaPaS assessment by the primary clinical teams showed a sensitivity of 83.7% and specificity of 100%.

**Table 4:** Comparison of PaPaS between the pediatric palliative care and primary clinical teams

Domain and item number	Palliative care team (mean, SD)	Primary clinical team (mean, SD)	*p*-value
≥1 year old			
Sum	22.0 (5.3)	20.4 (6.7)	< 0.001
Domain 1. Trajectory of disease and impact on daily activities of the child			
Q1.1 Trajectory of disease and impact on daily activities of the child	2.6 (1.2)	2.3 (1.4)	0.011
Q1.2 Increase of hospital admission	2.8 (0.7)	2.1 (1.4)	< 0.001
Domain 2. Expected outcome of treatment directed at the disease and burden of this treatment			
Q2.1 Treatment directed at the disease	1.1 (1.2)	1.4 (1.4)	< 0.001
Q2.2 Burden of treatment	2.8 (1.0)	2.5 (1.1)	< 0.001
Domain 3. Symptom and problem burden			
Q3.1 Symptom intensity or difficulty of symptom control	2.5 (1.1)	2.4 (1.3)	0.083
Q3.2 Psychological distress of patient related to symptoms	2.5 (1.0)	2.3 (1.1)	0.145
Q3.3 Psychological distress of parents or family related to symptoms and suffering of the child	2.9 (1.0)	2.7 (1.1)	0.073
Domain 4. Preferences/needs of patient or parents, preference of health professional			
Q4.1 Patient/parents wish to receive palliative care or formulated needs than are best met by palliative care	3.0 (1.8)	2.3 (2.0)	0.005
Q4.2 You/your team feel that this patient would benefit from palliative care	4.0 (0)	3.8 (0.9)	0.331
Domain 5. Estimated life expectancy			
Q5.1 Estimated life expectancy	0.6 (1.1)	0.7 (1.2)	0.348
Q5.2 “Would you be surprised if this child were to suddenly die in 6 months’ time?”	0.3 (0.7)	0.2 (0.6)	0.181
< 1 year old			
Sum	14.2 (4.0)	13.5 (4.5)	0.256
Domain 2. Expected outcome of treatment directed at the disease and burden of this treatment			
Q2.1 Trajectory of disease and impact on daily activities of the child	2.1 (1.5)	2.3 (1.6)	0.332
Domain 3. Symptom and problem burden			
Q3.1 Symptom intensity or difficulty of symptom control	3.2 (1.0)	2.9 (1.1)	0.096
Q3.3 Psychological distress of parents or family related to symptoms and suffering of the child	3.2 (1.0)	2.6 (1.1)	0.046
Domain 4. Preferences/needs of patient or parents, preference of health professional			
Q4.1 Patient/parents wish to receive palliative care or formulated needs than are best met by palliative care	3.5 (1.3)	2.1 (2.1)	0.009
Q4.2 You/your team feel that this patient would benefit from palliative care	4 (0)	4 (0)	n/a
Domain 5. Estimated life expectancy			
Q5.1 Estimated life expectancy	1.2 (1.5)	1.3 (1.6)	0.668
Q5.2 “Would you be surprised if this child were to suddenly die in 6 months’ time?”	0.9 (1.0)	0.7 (1.0)	0.588

*Abbreviation: PaPaS Scale* Paediatric Palliative Screening Scale, *SD* standard deviation

## Discussion

To our knowledge, this is the first study to compare the palliative care needs assessed by primary clinical and PPC teams using the PaPaS. We found that the PaPaS can be a useful tool to assess the palliative care needs of children with life-threatening illnesses by using consultation-based PPC services in tertiary hospitals in Korea.

Although it is ideal to deliver PPC through a shared care model in which the PPC team works together with primary care clinicians, there is still little consensus between the PPC and primary clinical teams who are not familiar with PPC services regarding the needs of children and their families [11, 13, 20]. In an earlier study that used this tool, Chong et al. reported the utility and feasibility of the PaPaS as a tool for screening admissions or determining the continuation of PPC after 1 year of home-based PPC service. This study demonstrated the transparency and reliability of the PaPaS through its objective and standardized scoring system, and suggested that widespread adoption of the PaPaS may minimize the gaps in communication, continuity of care, and collaboration across settings [7]. However, both the original author of the PaPaS and Chong’s studies were based on the PaPaS assessed by PPC specialists. The consistency of PaPaS assessment by primary care clinicians who are not familiar with PPC with the PPC team’s assessments has not been evaluated, considering that they may have considerably different perspectives and levels of understanding about PPC [7, 13, 14]. Therefore, we compared the palliative care needs assessed by primary clinical and PPC teams to evaluate the agreement between them. They showed moderate agreement and especially, medical specialists had a substantial agreement with PPC teams. In patients ≥1 year old, mean scores of five out of 11 questions in the PaPaS and mean total PaPaS scores were significantly lower in the referring clinical team, showing that the referring team tends to rate the overall palliative care need lower than the PPC team. However, the mean scores of both groups, regardless of medical specialists or residents, were exceeded the cut-off score (15 points), demonstrating that even assessments by clinicians who are less familiar with palliative care can pass the cut-off score. Individual questions that showed a statistical difference in the average scores were considered to have no clinical significance due to the marginal differences in values (i.e., 2.6 vs. 2.3 for Q1.1; and 1.1 vs. 1.4 for Q1.2).

Neonates and infants aged < 1 year have been excluded from previous studies due to different disease trajectories and needs. Since there were no validated tools in previous studies, we evaluated the palliative care needs of this age group using a modified PaPaS tool. As the mean total scores did not differ between the two groups and were higher than the cut-off value (10 points) in both groups, we expect it to be used as a preliminary tool since there are no verified tools for patients < 1 year old.

Furthermore, the PaPaS score correlated with the Lansky Performance status. The total PaPaS and Q1.1, which evaluated patient performance, scores demonstrated a meaningful correlation with the patient’s performance. Therefore, the PaPaS may reflect patients and their families’ needs by assessing the deterioration of patient performance.

The strength of this study includes evaluating the PaPaS of primary care clinicians for the purpose of the tool development. Second, we tried to validate the Korean version of PaPaS and this is the first study that evaluates the translated version of PaPaS. Third, we attempted to apply the tool to infants albeit this has limitations. Lastly, we compared PaPaS with the Lansky performance scale to assess the performance evaluation section of this tool. However, it is important to note some limitations. First, it was not a prospective study. Since PPC is still an unfamiliar model of care in South Korea due to a lack of understanding among the general public and health care providers, this study was performed as a retrospective analysis of PaPaS data derived from the national pilot project. For the same reason, the subjects were limited to patients referred to the PPC team. Furthermore, both teams assessed PaPaS in only 23.3% although primary care physicians were encouraged to assess patients with the PaPaS. Therefore, the results should be cautiously interpreted because of selection bias. Future studies including patients with complex diseases are needed, and policy makers and clinicians should continue to integrate palliative care to usual medical care. In addition, if the future studies can assess the association between the PaPaS and patients’ outcomes (eg. morbidity, mortality, PPC visits), potential benefits of the PaPaS can be shown. Third, the modified PaPaS used for neonates and children < 1 year old in this study has not been validated yet and the number of enrolled patients was too small for validation. For those < 1 year old, validation of the modified PaPaS in a larger study is required. Fourth, only one of five domains was compared with the Lansky performance scale. This may be the reason for the statistical significance in the correlation between the two groups, although not exceptionally strong. Because there were few tools assessing needs for PPC, it was difficult to validate the PaPaS externally. Future studies are needed for external validation of the PaPaS. Finally, this study was limited to consultation-based PPC services in a tertiary hospital setting. Understanding PPC and its availability may vary depending on health care systems, medical conditions, culture, priorities, and values for medical services; therefore, studies in various settings are required.

In conclusion, the PaPaS showed good agreement between primary care and palliative care teams, suggesting it may be a useful tool to assess patients and their families’ palliative care needs. Although the referring team tended to underestimate the palliative care needs compared to the PPC team, their PaPaS score was sufficient to identify the need for palliative care. Thus, the PaPaS as a tool could help primary care clinicians indentify the need and appropriate timing of referrals, especially in countries where PPC has been newly introduced.

## Data Availability

The datasets used and/or analyzed during the current study are available from the corresponding author on reasonable request.
